# Sex hormones shape EEG-based functional connectivity in early-stage Parkinson’s disease patients

**DOI:** 10.1016/j.nicl.2024.103721

**Published:** 2024-12-06

**Authors:** Matteo Conti, Roberta Bovenzi, Mariangela Pierantozzi, Clara Simonetta, Valerio Ferrari, Jacopo Bissacco, Rocco Cerroni, Claudio Liguori, Francesca Di Giuliano, Nicola Biagio Mercuri, Tommaso Schirinzi, Alessandro Stefani

**Affiliations:** a Neurology Unit, Department of Systems Medicine, University of Rome “Tor Vergata”, Rome, Italy; b UOSD Parkinson Centre, Tor Vergata University Hospital, Rome, Italy; c Neuroradiology Unit, Department of Biomedicine and Prevention, University of Rome “Tor Vergata”, Rome, Italy

**Keywords:** Parkinson’s disease, Functional connectivity, Resting-state network, HD-EEG, Sex hormones, Sex dimorphism

## Abstract

Parkinson’s disease (PD) epidemiology and clinical features are sexually dimorphic. However, there are no data based on EEG functional connectivity (FC). Likewise, the contribution of sex hormones on brain FC has never been evaluated. Here, we aimed to investigate the association between biological sex and sex hormones on cortical FC changes in PD using high-density EEG. This study involved 69 early-stage PD patients (F/M 27/42) and 69 age-matched healthy controls (HC) (F/M 30/39). Sex hormone levels (total-testosterone (TT), estradiol (E2), follicle-stimulating hormone (FSH), and luteinizing hormone (LH)) were assessed in PD patients. Data were recorded with a 64-channel EEG system. Source reconstruction method was used to identify brain activity. Cortico-cortical FC was analysed based on the weighted phase-lag index (wPLI) in θ-α-β-low γ bands. Network-based statistic (NBS) was used to compare FC between genders in HC and PD and to study the relationship between FC and sex hormones in PD. PD exhibited a hypoconnected network at θ and α bands and a hyperconnected network at β band compared to HC. Male HC showed a hyperconnected network at α-band compared to female HC. Conversely, males with PD showed a hypoconnected network at α-band compared to females with PD. In females and males with PD, E2 positively correlated with α-FC, while gonadotropins positively correlated with β-FC. TT positively correlated with the θ-FC only in males. Sex hormones shape EEG-FC in both males and females with PD, supporting their major influence on PD pathophysiology.

## Highlights

• Study examines sex and sex hormones’ impact on EEG functional connectivity in PD.
• 69 early-stage PD patients and 69 controls underwent cortico-cortical FC analysis.
• PD showed hypoconnectivity in θ and α bands, hyperconnectivity in β band vs. HC.
• Males with PD showed hypoconnectivity in α band compared to females.
• Estradiol, gonadotropins, and testosterone shaped FC in both male and female PD.

## 1 Introduction

Parkinson’s disease (PD) is the second most common neurodegenerative disorder, characterized by widespread deposition of α-synuclein-containing Lewy bodies (LBs) and primary loss of dopaminergic neurons of Substantia Nigra pars compacta (SNpc).

PD has a prominent sexual dimorphism (Cerri et al., 2019, Meoni et al., 2020). Indeed, PD is twice as prevalent in males, and males tend to have an earlier age of onset (Moisan et al., 2016). Male patients also display faster motor progression and greater involvement in cognitive function, reflecting a peculiar vulnerability to pathogenic events underlying PD (Picillo et al., 2017). On the other hand, wearing-off phenomena and dyskinesias occur earlier in women, and female PD patients more frequently bear with levodopa-induced motor complications, neuropsychiatric manifestations, and overall poorer quality of life (QoL) (Bovenzi et al., 2024a, Picillo et al., 2017). However, the biological bases of these peculiarities have not yet been clarified.

Sex hormones are likely to play a substantial role in the sex differences observed in PD because of their neuroactive properties (Cerri et al., 2019). Nevertheless, the contribution of sex hormones to neural activity has not been demonstrated in vivo. In preclinical models, estrogens have shown neuroprotective and pro-dopaminergic properties (Kompoliti, 2003). On the other hand, gonadotropins have been shown to promote neurodegenerative changes, favoring PD dementia (Bovenzi et al., 2024c, 2023c). Conversely, the role of androgens on PD remains to be investigated (Bourque and Di Paolo, 2022, Bovenzi et al., 2024b).

In this regard, functional connectivity (FC) could represent a valid tool for understanding these phenomena in PD patients better. Indeed, FC, based on different techniques such as functional magnetic resonance imaging (fMRI) or electroencephalography (EEG), can estimate the interactions between brain regions and has been extensively studied in PD(Conti et al., 2024, Conti et al., 2022a, 2022b), improving the understanding of the heterogeneous pathophysiology and showing to be an effective biomarker of disease progression (Yassine et al., 2022).

Sex differences were found to have an impact on brain FC in both HC (Zhang et al., 2018) and PD (De Micco et al., 2019). Nevertheless, while most studies are based on fMRI techniques, no data is available on EEG-FC in sex differences in PD. Furthermore, to date, the influence of sex hormones on brain FC in PD patients is scarcely known.

In this study, we thus aimed to investigate, using high-density (HD) EEG, the role of sex hormones in the sexual dimorphism of cortical FC alterations in PD.

## 2 Methods

### 2.1 Study population

In our study, we enrolled patients diagnosed with idiopathic PD in the early stages of the disease, followed up at the Parkinson Unit of the University of Rome “Tor Vergata” from June 2022 to December 2023. The inclusion criteria of the study were: 1) diagnosis of PD according to MDS criteria (Postuma et al., 2015); 2) stage of disease according to Hoehn & Yahr scale ≤ 2 (Goetz et al., 2004); 3) duration of disease < 48 months; 4) no treatment with Levodopa (LD naïve), to overcome its possible long-term effects (Zappia and Nicoletti, 2010).

The exclusion criteria were instead: 1) history of epilepsy or other neurological conditions that may cause alteration of EEG; 2) history of major psychiatric pathologies; 3) dementia, defined as Mini-Mental State Examination (MMSE) score < 25 (Folstein et al., 1975); 4) brain parenchymal lesions on brain MRI (e.g., brain tumors, strokes, abscesses, or other infectious conditions); 5) acute or chronic internal disorders; 6) a history of gynecological/prostate malignancy; 7) use of hormone therapy; 8) fertile age in females. We decided to exclude fertile women to keep the hormonal levels homogeneous within the female PD group and to avoid potential influences of the menstrual cycle.

We also enrolled a group of HC, who were similar in age and sex distribution to the patients and had no history of major neurological disorders and no exclusion criteria.

The study was conducted in agreement with the ethical principles of Helsinki and approved by the Policlinico Tor Vergata Ethical Committee (protocol number RS 16/17). Informed consent was obtained from all individual participants included in the study.

### 2.2 Clinical evaluation

All PD patients received a complete clinical evaluation on the same day as the EEG recording. Motor impairment was assessed using the Hoehn and Yahr scale (H&Y) (Goetz et al., 2004) and the MDS Unified Parkinson’s Disease Rating Scale part 3 (MDS-UPDRS III) (Goetz et al., 2008). Moreover, the motor subscores were calculated as the sum of the specific items of MDS-UPDRS-III (rigidity 3.3; bradykinesia 3.4–3.8, 3.14; tremor 3.15–3.18; gait/postural stability 3.9–3.13). Cognitive function was assessed through mini-mental state examination (MMSE)(Folstein et al., 1975) and Montreal cognitive assessment (MoCA) (Nasreddine et al., 2005). Non-motor symptoms of PD were evaluated by the non-motor symptoms scale (NMSS) (Chaudhuri et al., 2007) and the SCales for Outcomes in PArkinson’s disease − Autonomic Dysfunction (SCOPA-AUT) (Visser et al., 2004). All clinical evaluations were performed after overnight withdrawal from any dopaminergic drug. For all PD patients, the levodopa equivalent daily dose (LEDD, mg/day) for non-levodopa dopaminergic medications was calculated using the conventional formula. Informed consent was obtained from each participant in this study.

### 2.3 Sex hormones assay

Blood sex hormone levels, including total testosterone (TT), estradiol (E2), follicle-stimulating hormone (FSH), and luteinizing hormone (LH), were quantified in n = 49 PD patients (F/M 21/28), according to standard procedures (Bovenzi et al., 2023c). All blood samples were taken between 8 and 10 am, after overnight fastening, on the same day of the EEG recording and the clinical assessment, after overnight withdrawal from any dopaminergic drug.

### 2.4 EEG recording

HD-EEG data were recorded for 10 min at a sampling rate of 1024 Hz, band-pass filtered at 0.5–50 Hz using a 64-channel EEG system (EbNeuro BePlus-ProStandard). Electrodes were positioned according to the 10–10 International System (Nuwer, 2018). Impedance was kept below 5 kΩ. HD-EEG recording was performed in the eyes-closed (EC) resting state: the subjects were instructed to keep their eyes closed while staying awake for 5 min (Yassine et al., 2022). Then, reactivity to eyes opening and activation tests were performed to exclude the appearance of epileptiform elements in the EEG data.

In the PD group, the HD-EEG recording was performed after overnight withdrawal from the last administration of dopaminergic therapy. The same HD-EEG recording was obtained in HC.

### 2.5 FC analysis

We then proceeded to the primary FC analysis of the data collected. The data analyses were performed a priori and blind to the clinical examinations.

HD-EEG recordings were segmented into epochs of 30 s each for visualization purposes (Yassine et al., 2022). The first epoch of each recording was discarded. Then, we manually selected the first six consecutive low-artifact epochs (total 180 s) that were retained for the following analysis. The same method was used for PD patients and HC.

Independent component analysis (ICA) was used to remove the residual EEG artifacts (Hyvärinen and Oja, 2000). Then, we proceeded with EEG source localization (Conti et al., 2022b). To this purpose, all subjects underwent MRI, and personal T1-weighted MPRAGE MRI sequence and EEG data were co-registered through the identification of the same anatomical landmarks. Each MRI was segmented using FreeSurfer software (Fischl, 2012), obtaining a cortex surface of 5000 vertices. Then, the Boundary Element Method (BEM) (Jatoi et al., 2016) was used to solve the forward problem, while weighted minimum-norm estimation (wMNE) was used to solve the inverse problem (Grech et al., 2008), projecting the EEG signal to the 5000 cortical sources. The source activity was averaged into 68 brain regions using the Desikan–Killiany atlas (Desikan et al., 2006).

FC was calculated in source space using the weighted phase lag index (wPLI), a measure known to reduce conduction volume artifacts, noise artifacts, and bias from small samples (Hardmeier et al., 2014). Phase information from the preprocessed signals was computed using the Hilbert transform in θ (4–8 Hz), α (8–13 Hz), β (13–30 Hz), and low-γ (30–45 Hz) frequency bands. Dynamic FC matrices were computed between any pair of regions in the different EEG bands on segments of 1-second length, with an overlap of 50 %, according to Welch’s method. Of note, this method is not affected by the length of the displayed epochs (Jwo et al., 2021). Those matrices were averaged across time epochs to obtain static FC matrices.

EEG post-processing, source localization, and FC analysis were made using the Brainstorm toolbox (Tadel et al., 2011), combined with custom-written scripts for MATLAB R2023b. Other details of the methodology we used can be found in this paper of our group (Conti et al., 2023).

### 2.6 Statistical analysis

Differences in qualitative and quantitative variables between groups were tested using the Chi-Square Test and ANOVA test, respectively. Moreover, we analyzed the Spearman correlations between clinical features and sex hormone levels in female and male PD groups.

Differences in θ, α, β, and low-γ FC between female PD, male PD, female HC, and male HC were analyzed using the network-based statistic (NBS). NBS is a cluster-based statistical method used in numerous previous studies (Conti et al., 2024, Conti et al., 2024a, De Micco et al., 2021, Yassine et al., 2022) that has been shown to provide greater statistical power than standard univariate tests and traditional correction methods (Zalesky et al., 2010).

Specifically, in this study, we performed a one-way ANCOVA test edge-by-edge between the four groups (female PD, male PD, female HC, and male HC), considering age, disease duration, LEDD, and the most affected side as covariates. Next, we computed the size of the network whose edges had greater weights than the defined F threshold. We then performed a permutation test, randomly assigning all subjects into one of the four groups, maintaining each size of groups N-1 times, and computed maximum sizes of networks whose edges had bigger weights than the threshold, resulting in an empirical null distribution of maximum sizes. The number of permutations used in the present study was 5000. Then, we assigned the P value of the network with a fraction of the occurrence whose sizes were larger than the size of the network of the original assignment. Moreover, we defined the mean network connectivity (mNC) as the average connectivity of an NBS network. An ANOVA test with Tukey–Kramer post-hoc analysis was used to evaluate differences in mNC between each pair of groups.

Choosing the NBS threshold F represents an arbitrary parameter, even if control of the family-wise error rate (FWER) is guaranteed irrespective of the threshold choice. In our study design, we chose the smallest threshold F, which identified a significant NBS network (p < 0.05) with at least a “medium” effect size. This choice was made to avoid the inclusion of excessively large networks, which, while statistically significant, would have a negligible effect size, potentially masking the meaningful and relevant connections within the brain network. The effect size for a given threshold F was estimated using omega-squared (ω^2^), as in this case, the general linear model (GLM) used in NBS was ANCOVA(Watson Peter, 2018). An estimated effect size > 0.06 was considered “medium” (Watson Peter, 2018). Considering the sample size (N = 138), the number of groups (k = 4), and the number of covariates (Ncov = 4), the initial threshold F was 4.2. The incremental step was 0.1.

The significant differential networks were described in terms of nodes and links. Nodes refer to the individual components of the network, which in our case were the 68 cortical regions of interest (ROIs) defined by the Desikan-Killiany atlas, while links represent the interactions between each pair of nodes.

In case of significant effect in the one-way ANCOVA, post-hoc analysis of each degree of the selected network was performed using the Tukey–Kramer method to identify possible inter-sex (female PD vs. male PD and female HC vs. male HC) and intra-sex (female PD vs. female HC and male PD vs. male HC) differences in specific subnetworks between groups. Age and gender were considered confounding factors in the NBS.

Finally, we analyzed the partial Pearson correlations between mNC of disrupted PD networks and clinical features (age, disease duration, MDS-UPDRS total scores and subscores, MoCA, MMSE, total NMSS, and single domains, SCOPA-AUT) and sex hormone levels (E2, total TT, FSH, LH), in male and female PD groups. Age, disease duration, BMI, and most affected side were considered covariates. Moreover, we estimated the out-of-sample performance of the partial Pearson correlations analysis using bootstrapping (BS) as a cross-validation method. This resampling technique allowed us to assess the stability and generalizability of our correlation estimates by repeatedly sampling subsets of the data and simulating different training and testing datasets. The number of BS iterations we used was 1000. In the results, we provided a 95 % confidence interval (BS-95 %CI) of the BS distribution of correlation coefficients as an estimation of the robustness of our findings.

All statistical analyses were conducted using two-tailed tests, and a significance level of p < 0.05 was employed throughout the study. No statistical power calculation was conducted prior to the study. The sample size was based on the available data and our previous experience with the design of an FC study based on NBS. There were no missing data from the study variables.

### 2.7 Cross-validation algorithm

The NBS algorithm is based on a general linear model (GLM). Therefore, NBS does not allow for the evaluation of generalizability and reproducibility of the results. Thus, we evaluated the out-of-sample reliability of the NBS using a novel NBS tool called NBS-Predict(Serin et al., 2021). NBS-Predict employs suprathreshold edge selection in the training set in the outer loop to identify a connected component among the set of suprathreshold edges. The suprathreshold edge selection is identical to the original NBS method (more details on this method can be found in this paper (Serin et al., 2021)). The model for predictive analysis was Linear discriminant Analysis. The cross-validation (CV) algorithm we used in our study is the leave-one-out, an exhaustive CV method. Using NBS-Predict, we estimated the following parameters for each network: accuracy, precision, Cohen’s Kappa, Area Under Curve (AUC), sensitivity, and specificity.

The statistical analysis was performed using MATLAB 2023b, NBS (Zalesky et al., 2010), and NBS predict (Serin et al., 2021) toolboxes. Graphs were based on custom-written scripts on MATLAB 2023b and R (ggplot2 package).

## 3 Results

From an initial cohort of 156 subjects (78 PD patients and 76 HC), 69 PD patients (F/M 27/42) and 69 HC (F/M 30/39) matching the study criteria were included in the study.

The demographic and clinical characteristics of PD patients and HC are reported in Table 1. No significant differences emerged between PD female and male groups, except for the total SCOPA AUT score (p < 0.001).

**Table 1:** Clinical and demographic data of the study population.

	**Female PD** **(n = 27)**	**Male PD** **(n = 42)**	**Female HC** **(n = 30)**	**Male HC** **(n = 39)**	**p-value**
**Age (y)**	61.8 ± 9.0	62.8 ± 9.0	57.2 ± 11.8	60.1 ± 10.5	n.s.
**Disease duration (m)**	29.3 ± 19.5	27.5 ± 19.4	—	—	n.s.
**BMI**	24.9 ± 2.6	25.5 ± 3.8	—	—	n.s.
**Laterality (L/R)**	13/16	19/23	—	—	n.s.
**H&Y stage**	2.0 ± 0.6	1.8 ± 0.4	—	—	n.s.
**MDS-UPDRS-III**	29.2 ± 12.6	27.0 ± 10.6	—	—	n.s.
**Rigidity**	5.1 ± 2.7	5.3 ± 3.1	—	—	n.s.
**Bradykinesia**	14.4 ± 5.6	12.7 ± 5.1	—	—	n.s.
**Gait/postural**	3.5 ± 2.1	2.7 ± 1.9	—	—	n.s.
**Tremor**	2.8 ± 3.2	4.0 ± 3.7	—	—	n.s.
**Total LEDD (mg/die)**	110.3 ± 133.2	100.8 ± 138.7	—	—	n.s.
**MoCA**	27.4 ± 2.2	27.0 ± 2.2	—	—	n.s.
**MMSE**	28.9 ± 1.8	28.7 ± 1.6	—	—	n.s.
**NMSS**	34.8 ± 35.1	32.3 ± 27.4	—	—	n.s.
**Domain 3 − Mood**	9.8 ± 12.0	7.8 ± 10.1	—	—	n.s.
**SCOPA-AUT**	23.0 ± 15.8	13.1 ± 10.5	—	—	**0.003**

Data are expressed as mean ± standard deviation of variables. Statistical significance is marked in bold. Age is expressed in years. Disease duration is expressed in months. All patients were L-dopa-naïve.

The serum levels of sex hormones in the PD patients who underwent blood sampling are reported in Table 2. Female PD patients, compared to males, had lower E2 and TT levels (0.5 ± 0.5 vs. 28.6 ± 16.9, p < 0.001, and 26.5 ± 19.8 vs. 484.8 ± 182.9, p < 0.001, respectively), and higher gonadotropins levels (FSH 64.9 ± 26.5 vs. 8.6 ± 12.3, p < 0.001, and LH 22.6 ± 10.5 vs. 3.7 ± 2.9, p < 0.001, respectively).

**Table 2:** Age and sex hormone levels in PD fertile and post-menopausal females, and PD males.

	**Female PD** **(n = 21)**	**Male PD** **(n = 28)**	**p-Value**
**Age**	63.2 ± 9.7	61.2 ± 8.9	n.s.
**Estradiol (pg/ml)**	0.5 ± 0.5	28.6 ± 16.9	p < 0.001
**Total Testosterone (ng/dl)**	26.5 ± 19.8	484.8 ± 182.9	p < 0.001
**FSH (mUI/ml)**	64.9 ± 26.5	8.6 ± 12.3	p < 0.001
**LH (mUI/ml)**	22.6 ± 10.5	3.7 ± 2.9	p < 0.001

Data are expressed as mean ± standard deviation of variables. Age is expressed in years. FSH, follicle-stimulating hormone, and LH, luteinizing hormone.

No significant correlations were found between clinical features and sex hormone levels in female and male PD.

### 3.1 Differences in FC between female PD, male PD, female HC, and male^-^ HC

First, we performed NBS analysis to investigate differences in FC across the four groups (female PD, male PD, female HC, and male HC). 

**Fig. 1:**
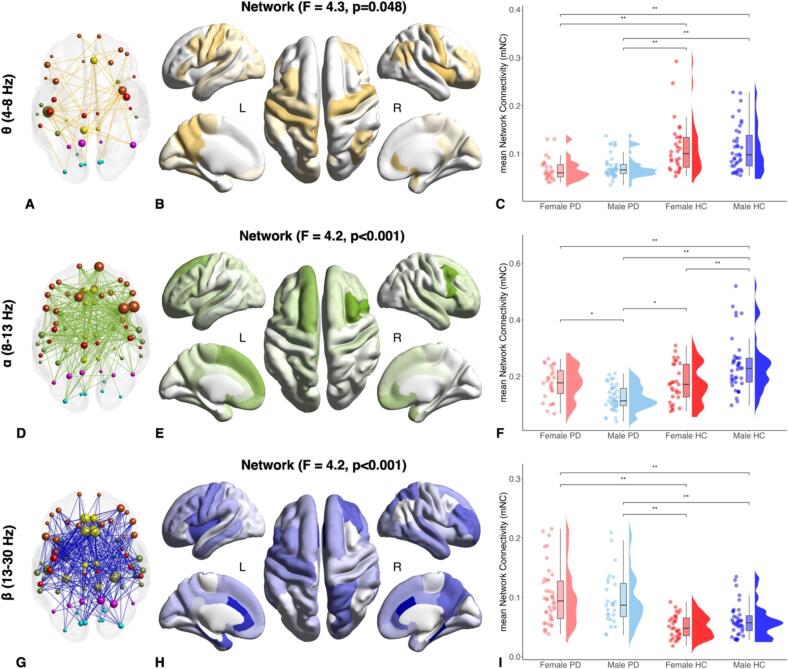
Graph representations of the differential networks between female PD, male PD, female HC, and male HC in θ (A), α (D), β (G) bands. Colors indicate different brain lobes: orange = fronto-insular, red = sensorimotor, yellow = limbic, green = temporal, magenta = parietal, light-blue = occipital; circle diameters are directly proportional to region of interest (ROI) degrees. Representation of ROI degrees on the model brain surface of the θ (B), α (E), β (H) differential networks. The mNC in female PD, male PD, female HC, and male- HC of the NBS differential networks in θ (C), α (F) and β (I) bands. Brain surface is based on MNI ICBM 152 non-linear template (Copyright (C) 1993–2009 Louis Collins, McConnell Brain Imaging Centre, Montreal Neurological Institute, McGill University).

We identified a statically significant network at θ band (F = 4.3, p = 0.048), where FC was significantly different between the four groups. The network was comprised of 38 nodes and 84 links and was rather lateralized in the left hemisphere (57.9 %). ROIs with higher degrees were part of the left dorsolateral prefrontal cortex, right insula, sensorimotor (bilateral precentral cortex), and limbic (manly right anterior cingulate area and bilateral posterior cingulate cortex) lobes *(*Fig. 1*, Panels A, B)*. The mNC was significantly lower in female PD compared to female HC (p < 0.001) and male HC (p < 0.001), and in male PD compared to female HC (p < 0.001) and male HC (p < 0.001) *(*Fig. 1*, Panel C)*.

We then identified a large FC network in the α band (65 nodes and 620 links, homogeneously distributed in the two hemispheres – 50.8 % left), which differed significantly between the four groups (F = 4.2, p < 0.001). ROIs with higher degrees were part of the dorsolateral, ventrolateral prefrontal cortex (left superior frontal, bilateral caudal middle frontal and pars opercularis ROIs), orbitofrontal cortex (bilateral medial and lateral orbitofrontal areas), sensorimotor (mostly left paracentral and precentral, and right postcentral cortex), and temporal (predominantly left superior temporal and right temporal pole) *(*Fig. 1*, Panels D, E)*. The mNC was significantly higher in male HC compared to male PD (p < 0.001), female PD (p = 0.001), and female HC (p = 0.001). Finally, the mNC was significantly higher in female PD compared to male PD (p = 0.015) *(*Fig. 1*, Panel F)*.

Finally, we identified a significant large network in the β band (t = 2.5, p = 0.030), showing differences in FC among the groups. The network was composed of 67 nodes and 612 connections and was equally distributed between the two hemispheres (left 50.1 %). Brain regions with higher degrees were part of the sensorimotor (mainly bilateral precentral and postcentral), limbic (predominantly bilateral anterior cingular cortex), left insula, and parietal (mostly right precuneus and superior parietal cortex) lobes *(*Fig. 1*, Panels G, H)*. The mNC was significantly higher in female PD compared to female HC (p < 0.001) and male HC (p < 0.001), and in male PD compared to female HC (p < 0.001) and male HC (p < 0.001) *(*Fig. 1*, Panel I)*.

No significant differences were observed in the other bands.

### 3.2 Inter-sex FC differences in HC and in PD cohorts

At this point, we analyzed EEG-FC differences between females and males, first in HC and then in PD patients, using a post-hoc analysis of the differential networks found.

**Fig. 2:**
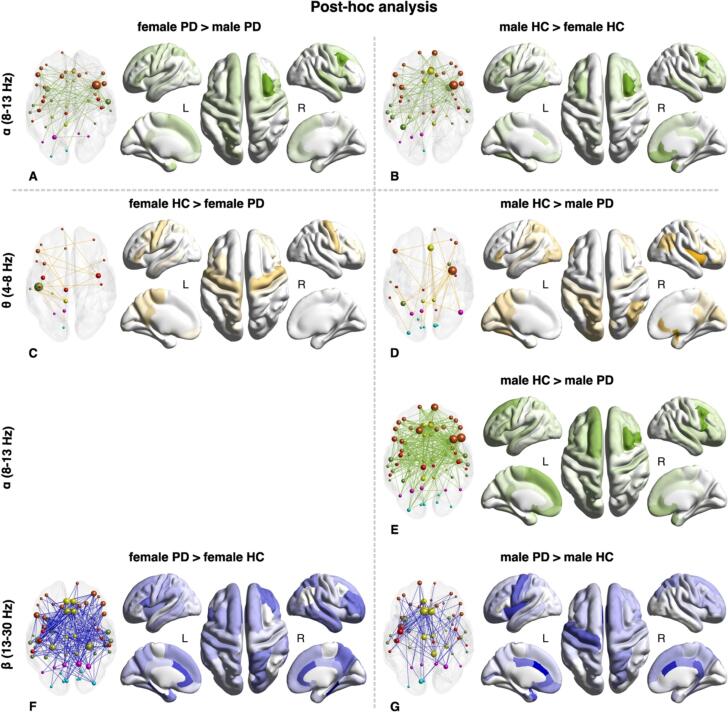
Graph representations (on the left of each panel) and representation of ROI degrees on the model brain surface (on the right of each panel) of the differential subnetwork between female and male PD (FC females > males) in α band (A); female and male HC (FC males > females) in α band (B); female HC and female PD in θ band (FC HC > PD) (C) and in β band (FC PD > HC) (F); male HC and male PD in θ band (FC HC > PD) (D), in α band (FC HC > PD) (E), and in β band (FC PD > HC) (G);. Colors indicate different brain lobes: orange = fronto-insular, red = sensorimotor, yellow = limbic, green = temporal, magenta = parietal, light-blue = occipital; circle diameters are directly proportional to region of interest (ROI) degrees. Brain surface is based on MNI ICBM 152 non-linear template (Copyright (C) 1993–2009 Louis Collins, McConnell Brain Imaging Centre, Montreal Neurological Institute, McGill University).

Among HC, we found a statistically significant subnetwork at the α band characterized by higher connectivity in males than females. This subnetwork was composed of 55 nodes and 248 links (40 % of the original network) and was slightly lateralized in the right hemisphere (59.2 %). The higher degrees ROIs of this network were part of the dorsolateral prefrontal cortex (bilateral caudal middle frontal ROIs), right orbitofrontal cortex, temporal lobe (mainly right temporal pole and bilateral middle and inferior temporal cortex), and limbic areas (primarily bilateral anterior cingulate cortex) *(*Fig. 2*, Panel B).* No further differences were observed between the two sexes.

Regarding PD patients, we observed a significant subnetwork in the α band, where FC was higher in PD females than males. This network comprised 47 nodes and 122 links (18.1 % of the original α network). ROIs with higher degrees belonged mainly to the left hemisphere (59.6 %) and were composed of the dorsolateral and dorsoventral prefrontal cortex (bilateral superior frontal and right caudal middle frontal areas), right sensorimotor (mostly bilateral and postcentral cortex) and left temporal pole *(*Fig. 2*, Panel A)*. No significant different subnetworks were found between PD females and males in the other frequencies*.*

### 3.3 Intra-sex FC differences between PD and HC cohorts

Then, we analyzed FC differences between female PD and HC and between male PD and HC, using post-hoc analysis of the differential networks found in the θ. α and β bands.

When analyzing females, we found a statistically significant subnetwork at the θ band, where the connectivity was lower in female PD compared to female HC. This subnetwork was composed of 20 nodes and 40 links (47.6 % of the original network) and was lateralized in the left hemisphere (82.5 %). The higher degrees ROIs of this subnetwork were part of the sensorimotor areas (mostly bilateral precentral cortex) and left transverse temporal cortex *(*Fig. 2*, Panel C).* On the contrary, we observed a significant subnetwork in the β band, where FC was higher in PD females compared to HC females. This subnetwork comprised 63 nodes and 460 links, including 75.2 % of the original links (460/612), and was equally distributed between the two hemispheres. Brain regions with higher degrees mainly belonged to the limbic lobe (predominantly bilateral anterior cingular cortex) *(*Fig. 2*, Panel F)*.

Regarding males, we identified a statistically significant subnetwork at the θ band, where the connectivity was lower in male PD compared to male HC. This subnetwork was composed of 26 nodes and 48 links (57.1 % of the original network) and was slightly lateralized in the right hemisphere (54.2 %). The higher degrees ROIs of this subnetwork were part of the right insula and right anterior cingulate *(*Fig. 2*, Panel D).* Moreover, we observed a significant subnetwork in the α band, where FC was higher in HC males compared to PD males. This network comprised 64 nodes and 602 links, largely overlapped with the original network, including 97.1 % of its links (602/620) *(*Fig. 2*, Panel E).* Finally, a significant subnetwork was found in the β band, where FC is higher in male PD compared to male HC. This subnetwork was composed of 52 nodes and 194 edges (31.7 % of the original network) and was slightly lateralized in the left hemisphere (58.8 %). ROIs with higher degrees were mainly part of the limbic lobe (bilateral anterior cingulate) and sensorimotor areas (mostly left precentral and postcentral cortex) *(*Fig. 2*, Panel G)*.

### 3.4 Cross-validation of the NBS analysis

We analyzed the out-of-sample reliability and prediction performance of the NBS network found in the above analyses using a cross-validation leave-one algorithm. Accuracy, precision, Cohen’s κ, AUC, sensitivity, and specificity of the differential NBS network in discriminating inter-sex (female PD vs. male PD and female HC vs. male HC) and intra-sex (female PD vs. female HC and male PD vs. male HC) differences are reported in Table 3.

**Table 3:** Cross-validation of the differential NBS network in inter-sex (female PD vs. male PD and female HC vs. male HC) and intra-sex (female PD vs. female HC and male PD vs. male HC) conditions based on NBS-Predict leave-one-out method. The parameters reported are in the first row the value of accuracy, precision, Cohen’s Kappa, AUC, sensitivity and specificity, and in the second row their relative 95% confidence interval (95%CI).

**Networks**	**Accuracy**	**Precision**	**Cohen’s κ**	**AUC**	**Sensitivity**	**Specificity**
**Female PD vs. Male PD**						
α − female > male	0.73 [0.70 0.74]	0.65 [0.58 0.71]	0.47 [0.25 0.68]	0.73 [0.59 0.84]	0.74 [0.54 0.89]	0.74 [0.58 0.85]
**Female HC vs. Male HC**						
α – female < male	0.69 [0.68 0.71]	0.74 [0.68 0.82]	0.41 [0.19 0.63]	0.69 [0.55 0.81]	0.67 [0.48 0.83]	0.74 [0.58 0.86]
**Female PD vs. female HC**						
θ − PD < HC	0.74 [0.73 0.77]	0.77 [0.70 0.86]	0.54 [0.32 0.76]	0.81 [0.67 0.90]	0.74 [0.54 0.88]	0.80 [0.62 0.92]
β − PD > HC	0.75 [0.68 0.76]	0.93 [0.82 1.00]	0.84 [0.72 0.91]	0.84 [0.72 0.91]	0.62 [0.46 0.75]	0.93 [0.77 1.00]
**Male PD vs. Male HC**						
θ – PD < HC	0.76 [0.75 0.78]	0.77 [0.70 0.87]	0.55 [0.37 0.73]	0.79 [0.67 0.87]	0.81 [0.67 0.91]	0.74 [0.58 0.87]
α – PD < HC	0.79 [0.78 0.81]	0.83 [0.75 0.90]	0.63 [0.46 0.80]	0.86 [0.76 0.81]	0.81 [0.67 0.91]	0.82 [0.67 0.92]
β − PD > HC	0.75 [0.72 0.76]	0.68 [0.61 0.75]	0.51 [0.30 0.72]	0.77 [0.62 0.87]	0.78 [0.58 0.91]	0.74 [0.59 0.86]

### 3.5 Association between dysfunctional networks and clinical scores in PD females and males

We thus analyzed the correlations between clinical scores and the mNC of the differential networks in female and male PD patients. Each correlation was corrected for age, disease duration, and most affected side of the disease, using Pearson’s partial correlation analysis.

**Fig. 3:**
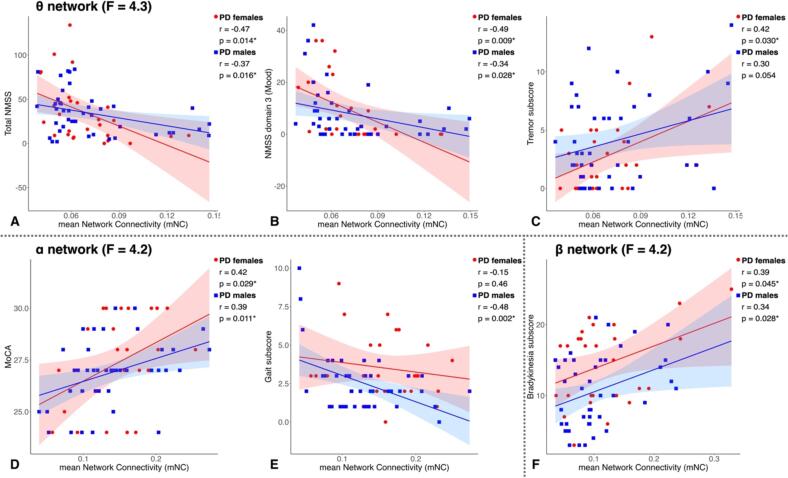
Correlations between the mNC of the differential θ network and total NMSS (A), NMSS domain 3 (B), and tremor subscore of MDS-UPDRS III (C) in PD females and males. Correlations between the mNC of the differential α network and MoCA (D), and gait subscore of MDS-UPDRS III (E) in PD females and males. Correlations between the mNC of the differential β network and bradykinesia subscore of MDS-UPDRS III (F) in PD females and males.

We found significant negative correlations between the mNC of the θ network (F = 4.3) and total NMSS in both PD females (r =  − 0.47, p = 0.014, BS-95 %CI [-0.67––0.30]), and PD males (r =  − 0.37, p = 0.016, BS-95 %CI [-0.56––0.16]) *(*Fig. 3*, Panel A)* and NMSS domain 3 (Mood) in female (r =  − 0.49, p = 0.009, BS-95 %CI [-0.66––0.32]), and male PD (r =  − 0.34, p = 0.028, BS-95 %CI [-0.49––0.15]) *(*Fig. 3*, Panel B)*. Moreover, we observed a significant positive correlation between the mNC of the θ network and the tremor subscore of the MDS-UPDRS-III in female PD (r = 0.42, p = 0.030, BS-95 %CI [-0.01 0.67]), while among male PD patients, the same correlation was at the limit of significance (r = 0.30, p = 0.054, BS-95 %CI [-0.07 0.60]) *(*Fig. 3*, Panel C).*

Regarding the α network (F = 4.2), we discovered a significant positive correlation between the mNC and the MoCA score in both female (r = 0.41, p = 0.033, BS-95 %CI [0.12 0.67]) and male PD (r = 0.39, p = 0.011, BS-95 %CI [0.11 0.59]) *(*Fig. 3*, Panel D).* Furthermore, we found a significant negative correlation between the α network mNC and the gait subscore of the MDS-UPDRS-III in male PD (r =  − 0.48, p = 0.002, BS-95 %CI [-0.67––0.19]), but not in female PD (r =  − 0.15, p = 0.46, BS-95 %CI [-0.47 0.19]) *(*Fig. 3*, Panel E).*

Finally, we observed significant positive correlations between the mNC of the β network and the bradykinesia subscore the MDS-UPDRS-III in both female (r = 0.39, p = 0.045, BS-95 %CI [-0.12 0.65]) and male PD (r = 0.34, p = 0.028, BS-95 %CI [0.11 0.55]).

No significant correlations were found between FC networks and age, disease duration, MMSE, SCOPA-AUT, total MDS-UPDRS-III, or the rigidity subscore.

### 3.6 Association between dysfunctional networks and sex hormones in PD females and males

**Fig. 4:**
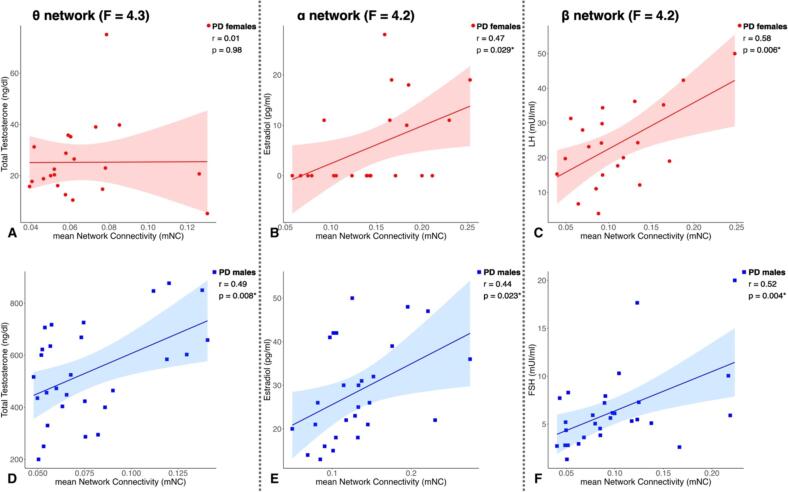
Correlations between the mNC of the differential θ network and the total testosterone levels in PD females (A) and males (D). Correlations between the mNC of the differential α network and the estradiol levels in PD females (B) and males (E). Correlations between the mNC of the differential β network and the LH levels in PD females (C) and the FSH levels in PD males (F).

In the female PD group, we found a significant positive correlation between the mNC of the differential α network (F = 4.2) and the estradiol levels (r = 0.47, p = 0.029, BS-95 %CI [0.17 0.74]) (Fig. 4*, Panel B*). Moreover, we observed a significant positive correlation between the mNC of β FC network (F = 4.2) and LH levels (r = 0.58, p = 0.006, BS-95 %CI [0.09 0.81]) (Fig. 3*, Panel C*). A positive correlation was also found between the mNC of β FC network and FSH levels, which, however, did not reach statistical significance (r = 0.33, p = 0.16, BS-95 %CI [-0.21 0.72]).

In the PD male group, we observed a positive correlation between the mNC of the differential θ network (F = 4.3) and total testosterone levels (r = 0.49, p = 0.008, BS-95 %CI [0.13 0.69]) (Fig. 3*, Panel D*). Moreover, we discovered a significant positive correlation between the mNC of the α FC network (F = 4.2) and the estradiol levels (r = 0.44, p = 0.023, BS-95 %CI [0.13 0.71]) (Fig. 3*, Panel D*). Additionally, we found a significant positive correlation between the mNC of the β FC network (F = 4.2) and FSH levels (r = 0.52, p = 0.004, BS-95 %CI [0.10 0.79]) (Fig. 3*, Panel E*). No significant correlation was observed between the mNC of the β FC network and the LH levels (r = 0.068, p = 0.77, BS-95 %CI [-0.24 0.37]).

No other significant results were found between sex hormone levels and the mNC of PD dysfunctional networks.

## 4 Discussion

This study, by means of the HD-EEG, demonstrated that FC differed between females and males in both early-stage levodopa naïve PD patients and healthy controls and was diversely associated with clinical features and sex hormone levels in patients depending on the sex, providing substantial background for the well-known sexual dimorphism of the disease.

Our group recently described the EEG-based FC features characterizing PD with respect to HC (Conti et al., 2023). Current data confirm those findings on a larger sample, showing how early-stage both female and male PD patients may present with band- and network-specific functional changes compared to female and male HC. In particular, here, both female and male PD patients stood out for a reduced FC in θ bands, mainly in prefrontal, insular, and sensorimotor areas, and an increased FC in β band, predominantly in the sensorimotor, limbic, and parietal areas, compared to both female and male HC. On the contrary, a reduced FC in the α band, mainly in prefrontal, sensorimotor, temporal, and limbic regions, was observed only in male PD patients compared to male HC, while no significant differences were found between female PD patients and female HC.

When analyzing EEG-FC changes according to sex, either in patients or HC, we noticed a sex-dimorphic network in the α band in both groups. Specifically, the EEG-FC network in the α band was increased in male HC compared to females, primarily in dorsolateral prefrontal, orbitofrontal, temporal, and limbic areas. Conversely, the same network was more disrupted in male PD patients compared to females, mostly in the left hemisphere, within the dorsolateral and ventral prefrontal cortex, the sensorimotor areas, the left insula, and the temporal lobe. Albeit based on different techniques, our findings are in line with compelling evidence from fMRI studies. In fact, Zhang et al., utilizing the data from 494 HC of the Human Connectome Project, found that healthy males can exhibit a widespread greater FC than females, mostly in frontal, parietal, and temporal lobes, and that the male brain can be more segregated while the female brain more integrated(Zhang et al., 2016). Otherwise, the FC can increase in the sensorimotor network in healthy males compared to females(Weis et al., 2019). Also fMRI-based studies on PD disclosed a higher FC in female patients compared to males, mostly involving the sensorimotor areas (De Micco et al., 2018).

Patterns of EEG-FC were also variously associated with main clinical features in patients, either in a sex-specific or a sex-independent manner.

The mNC of θ dysfunctional network, which predominantly involved the prefrontal areas, negatively correlated to total NMSS and NMSS domain 3 (mood) in both sexes. As the EEG θ activity might mark the emotional processes and the functional impairment within the dorsolateral-prefrontal cortex might account for depression in PD(Wen et al., 2013), we could suppose that the prefrontal-limbic dysfunction of θ FC reflects the impairment of emotional process and the development of mood disorder in PD, regardless of the sex.

Testosterone seems to have a role in mediating this association, as the positive relationship between EEG-FC at θ band and total testosterone levels in male patients suggests. In a previous study, we demonstrated that testosterone might contribute to male vulnerability to PD, favoring some neuropathological changes, including the accumulation of synucleinopathy and basal ganglia atrophy (Bovenzi et al., 2023c). Otherwise, testosterone modulates mood and behavior, and lower levels have been found in male PD patients with depression, anxiety, and anhedonia (Okun et al., 2002). Here, the association between hormone and FC at θ band could be thus referred to as the latter property of the hormone, confirming its capability to affect the neuropsychological circuits of the male brain. The absence of correlations in female patients could rather depend on the low circulating hormone levels, which can prevent the statistical significance.

The θ mNC also positively correlated with the MDS-UPDRS III tremor subscore in PD females, whereas, in males, the correlation did not reach statistical significance. Consistent with our results, an increased synchronization in oscillatory activity between the STN, the GPi, the thalamus, and the motor cortex, which occurs at the resting tremor frequency θ (4–7 Hz), has been documented in PD in studies, and predominantly in those with tremor-dominant forms (Brown, 2003). Such synchronization seems to be linked to the activation of GPi-thalamic-cortical pathways with pro-tremorgenic activity.

The mNC of the α network, which was significantly hypoconnected in prefrontal, sensorimotor, and temporal regions of PD patients, directly correlated with cognition, as assessed by the MoCA scores, in both sexes. The reduction of EEG-FC at α band is commonly observed in PD at different disease stages (Conti et al., 2023, Yassine et al., 2022) and in other neurodegenerative disorders, such as Alzheimer’s disease (AD) or Lewy body disease (LBD) (Babiloni et al., 2019). The biological substrate is thought to be the disruption of ascending and cortical cholinergic systems (Babiloni et al., 2019). The α-FC decrease in PD patients’ cortical areas could thus reflect a subcortical and/or cortical cholinergic impairment, leading to cognitive deterioration since the earliest stages of the disease (van der Zee et al., 2022).

We also found that the mNC of the α network had a male-specific direct correlation with the MDS-UPDRS-III gait/postural stability subscore. A reduction of synchronization in the α band between the pedunculopontine nucleus and motor cortex has already emerged in PD and atypical parkinsonisms (Stefani et al., 2007), suggesting the alteration in cholinergic neurotransmission as a potential mechanism underlying gait dysfunction in PD (Morris et al., 2019). It is well-known that male PD patients can suffer from axial postural abnormalities, faster motor progression, and cognitive impairment more frequently than females (Picillo et al., 2022). Therefore, we propose that male patients had a selective susceptibility to cholinergic transmission within the frontotemporal areas, which may account for their predisposition to a worse motor phenotype.

This hypothesis is partially supported by the correlation analysis with sex hormones. In fact, in both the sexes, the EEG-FC at α band positively correlated with E2 levels. Estrogen levels, mostly E2, dramatically decrease in post-menopausal females, produced by a few extragondal sites rather than the ovary (Hall, 2015). In males, a small number of estrogens is synthesized by the aromatization of androgens in peripheral tissues. We have already demonstrated that E2 levels can significantly affect motor features in PD patients, regardless of sex (Bovenzi et al., 2023a, Bovenzi et al., 2023b, 2023c). Therefore, the positive association between FC at α band and E2 levels indicates that estrogens might preserve motor brain circuit integrity, in particular, the dopaminergic and the basal forebrain cholinergic ones, regardless of the sex, in line with evidence from animal models (Bourque et al., 2023). Accordingly, we could hypothesize that the higher lifetime exposure to estrogens in female patients might protect neural circuits related to α-FC; conversely, the lower estrogenic tone and the detrimental effects of testosterone might account for the greater vulnerability of the male patients.

Finally, consistent with our previous work (Conti et al., 2023), we observed a positive correlation between the mean connectivity of the β dysfunctional network FC and the MDS-UPDRS III bradykinesia subscore in female and male PD patients. The increased synchronization in the β band between basal ganglia (BG) and motor cortex is a typical neurophysiological marker of PD(Little and Brown, 2014), classically related to the bradykinesia severity (Brown, 2003). Indeed, the balance between β and γ oscillation in the motor cortex plays a relevant role in modulating movement performance in PD, with β oscillations favoring bradykinesia, whereas γ cortical oscillations have an anti-bradykinetic effect (Guerra et al., 2022). However, our data show that the increase of β-FC at the cortical level can also involve extra motor areas, including limbic and parietal regions, which suggests that β network abnormalities may underlie other, non-purely motor features. Here, the gonadotropin levels – FSH in PD males and LH in PD post-menopausal females – directly correlated with the increased β FC in those areas. Circulating gonadotropins increase with aging in both sexes, reaching the highest levels in post-menopausal females in response to the estrogenic decline. In PD patients, both males and post-menopausal females, such an increase has been associated with a significant burden of brain amyloidopathy and a major occurrence of cognitive complaints. Since bradykinesia in PD might also imply a cognitive contribution (Bologna et al., 2020), we can interpret the reciprocal correlations among β FC, bradykinesia, and gonadotropin levels as a sex-independent and age-related trajectory (Bovenzi et al., 2023c), leading to greater neurodegeneration and higher risk for cognitive decline.

This study has some limitations, including the cross-sectional design, the partial data from the control group, and, although all the patients were LD-naïve, the potential confounding effect of other dopaminergic medications on either FC or sex hormone production. Moreover, we must acknowledge the low spatial resolution of the EEG technique and the difficulty in investigating the high-γ band because of the muscle artifacts. However, we enrolled a homogeneous population, strictly selected to avoid any biases due to comorbidity or concurrent conditions, and we also performed statistical analyses adjusting for main potential confounding factors. Actually, there were no significant differences between PD patients and HC in age, and the analyses were also covariate for this potential confounding factor, which is a well-known factor affecting FC (Varangis et al., 2019). The PD group included patients with similar disease duration and pharmacological treatments without major cognitive impairment, assessed after overnight withdrawal from any dopaminergic drug discontinuation to avoid potential effects on both the FC-EEG and the hormone levels. Indeed, no significant relationships were found between FC changes and disease duration in this study. Larger, prospective studies, including patients at various disease stages, are needed to investigate any sex-dimorphic trajectory of FC changes. Moreover, although the sex hormone levels were not assessed in the HC, the sex hormone levels in the PD cohort were within normal ranges as expected by sex and age, despite conflicting evidence regarding an altered hormone profile in PD (Nitkowska et al., 2015). Moreover, a limitation of the study lies in the modest strength of the observed correlations, which, although statistically significant, could be influenced by floor or ceiling effects in the raw data, such as MoCA scores or hormone levels. However, a bootstrap method was used to estimate the out-of-sample reliability of the found correlations. Again, future studies should consider measuring sex hormone levels in a control group to better clarify the extent to which the observed phenomena in the present study are due to sex, hormone levels, the disease, or rather potential interactions between these factors. Finally, we observed lateralized dysfunctions of specific ROI of one hemisphere compared to the other. However, we have not related these differences to the more affected side of the disease of PD patients in the present study. Nevertheless, we considered the more affected side as a covariate in the NBS analysis.

## 5 Conclusions

This study demonstrated that the biological sex and the sex hormone levels can account for different network- and band-specific FC features in early-stage PD patients, which in turn may underly, at least in part, the dimorphic clinical presentation of the disease. The main intersex difference was in the α band-FC, a network basically sustained by the cholinergic signaling, which was more disrupted in males than in females. The network integrity was directly correlated with E2 levels in both sexes, suggesting a protective effect of the estrogens on those neural circuits per se. However, the greater lifetime exposure to estrogens might justify the sex-specific α band-FC preservation. Otherwise, the male-specific α band-FC impairment might represent the substrate for axial and gait disturbances or cognitive issues, which are more frequent in male patients. Male patients also had a direct association between testosterone levels and the θ band FC, whose integrity tends to decrease in PD, favoring depression and apathy occurrence, which indicates the hormone’s capability, at the high male-specific circulating levels, to stimulate mood and behavior circuits. Finally, a direct correlation resulted between gonadotropins, whose levels physiologically increase with aging and menopause, and the FC in the β band, which may track an age-related and sex-independent neurodegenerative trajectory responsible for bradykinesia and eventual cognitive decline, two peculiar features of elderly.

These findings, although needing appropriate replication and confirmation on a larger scale, support the central role of sex hormones in driving PD pathophysiology by direct and novel human-based evidence and even provide hints for personalized neuromodulation-based therapeutic approaches.

## CRediT authorship contribution statement

**Matteo Conti:** Writing – original draft, Software, Methodology, Formal analysis, Data curation, Conceptualization. **Roberta Bovenzi:** Writing – original draft, Formal analysis, Data curation, Conceptualization. **Mariangela Pierantozzi:** Visualization. **Clara Simonetta:** Resources, Data curation. **Valerio Ferrari:** Data curation. **Jacopo Bissacco:** Data curation. **Rocco Cerroni:** Visualization. **Claudio Liguori:** Visualization. **Francesca Di Giuliano:** Validation. **Nicola Biagio Mercuri:** Visualization. **Tommaso Schirinzi:** Writing – review & editing, Supervision, Conceptualization. **Alessandro Stefani:** Writing – review & editing, Supervision, Conceptualization.

## Declaration of competing interest

The authors declare that they have no known competing financial interests or personal relationships that could have appeared to influence the work reported in this paper.

## Data Availability

Data will be made available on request.

## References

[b0005] Babiloni, C., Del Percio, C., Pascarelli, M.T., Lizio, R., Noce, G., Lopez, S., Rizzo, M., Ferri, R., Soricelli, A., Nobili, F., Arnaldi, D., Famà, F., Orzi, F., Buttinelli, C., Giubilei, F., Salvetti, M., Cipollini, V., Franciotti, R., Onofrj, M., Stirpe, P., Fuhr, P., Gschwandtner, U., Ransmayr, G., Aarsland, D., Parnetti, L., Farotti, L., Marizzoni, M., D’Antonio, F., De Lena, C., Güntekin, B., Hanoğlu, L., Yener, G., Emek-Savaş, D.D., Triggiani, A.I., Taylor, J.P., McKeith, I., Stocchi, F., Vacca, L., Hampel, H., Frisoni, G.B., De Pandis, M.F., Bonanni, L., 2019. Abnormalities of functional cortical source connectivity of resting-state electroencephalographic alpha rhythms are similar in patients with mild cognitive impairment due to Alzheimer’s and Lewy body diseases. Neurobiol Aging 77. doi: 10.1016/j.neurobiolaging.2019.01.013. 10.1016/j.neurobiolaging.2019.01.013 30797169

[b0010] Bologna M., Paparella G., Fasano A., Hallett M., Berardelli A. (2020). Evolving concepts on bradykinesia. Brain.

[b0015] Bourque M., Di Paolo T. (2022). Neuroactive steroids and Parkinson’s disease. Curr. Opin. Endocr. Metab. Res..

[b0020] Bourque M., Morissette M., Soulet D., Di Paolo T. (2023). Impact of sex on neuroimmune contributions to Parkinson’s disease. Brain Res. Bull..

[b0025] Bovenzi R., Conti M., Degoli G.R., Cerroni R., Artusi C.A., Pierantozzi M., Stefani A., Mercuri N.B., Schirinzi T. (2023). Pregnancy, fertile life factors, and associated clinical course in PRKN early-onset Parkinson’s disease. Neurol. Sci..

[b0030] Bovenzi R., Conti M., Degoli G.R., Cerroni R., Simonetta C., Liguori C., Salimei C., Pisani A., Pierantozzi M., Stefani A., Mercuri N.B., Schirinzi T. (2023). Shaping the course of early-onset Parkinson’s disease: insights from a longitudinal cohort. Neurol. Sci..

[b0035] Bovenzi R., Sancesario G.M., Conti M., Grillo P., Cerroni R., Bissacco J., Forti P., Giannella E., Pieri M., Minosse S., Ferrazzoli V., Pucci N., Laudazi M., Floris R., Garaci F., Pierantozzi M., Stefani A., Mercuri N.B., Picchi E., Di Giuliano F., Schirinzi T. (2023). Sex hormones differentially contribute to Parkinson’s disease in males: a multimodal biomarker study. Eur. J. Neurol..

[b0040] Bovenzi R., Conti M., De Franco V., Pierantozzi M., Schirinzi T., Cerroni R., Stefani A., Mercuri N.B., Liguori C. (2024). Sex differences in Parkinson’s disease-related non motor symptoms: a focus on sleep problems. Acta Neurol. Belg..

[b0045] Bovenzi R., Conti M., Simonetta C., Bissacco J., Mascioli D., Michienzi V., Pieri M., Cerroni R., Liguori C., Pierantozzi M., Stefani A., Mercuri N.B., Schirinzi T. (2024). Contribution of testosterone and estradiol in sexual dimorphism of early-onset Parkinson’s disease. J. Neural Transm..

[b0050] Bovenzi R., Schirinzi T., Conti M., Sancesario G.M., Zenuni H., Simonetta C., Bissacco J., Mascioli D., Pieri M., Cerroni R., Stefani A., Mercuri N.B., Pierantozzi M. (2024). A biological characterization of patients with postmenopausal Parkinson’s disease. J. Neurol..

[b0055] Brown P. (2003). Oscillatory nature of human basal ganglia activity: Relationship to the pathophysiology of parkinson’s disease. Mov. Disord..

[b0060] Cerri S., Mus L., Blandini F. (2019). Parkinson’s Disease in Women and Men: What’s the Difference?. J. Parkinsons Dis..

[b0065] Chaudhuri K.R., Martinez-Martin P., Brown R.G., Sethi K., Stocchi F., Odin P., Ondo W., Abe K., MacPhee G., MacMahon D., Barone P., Rabey M., Forbes A., Breen K., Tluk S., Naidu Y., Olanow W., Williams A.J., Thomas S., Rye D., Tsuboi Y., Hand A., Schapira A.H.V. (2007). The metric properties of a novel non-motor symptoms scale for Parkinson’s disease: results from an international pilot study. Mov. Disord..

[b0070] Conti M., Bovenzi R., Garasto E., Schirinzi T., Placidi F., Mercuri N.B., Cerroni R., Pierantozzi M., Stefani A. (2022). Brain functional connectivity in de novo Parkinson’s disease patients based on clinical EEG. Front. Neurol..

[b0075] Conti M., Stefani A., Bovenzi R., Cerroni R., Garasto E., Placidi F., Liguori C., Schirinzi T., Mercuri N.B., Pierantozzi M. (2022). STN-DBS induces acute changes in β-band cortical functional connectivity in patients with Parkinson’s disease. Brain Sci..

[b0080] Conti M., Guerra A., Pierantozzi M., Bovenzi R., D’Onofrio V., Simonetta C., Cerroni R., Liguori C., Placidi F., Mercuri N.B., Di Giuliano F., Schirinzi T., Stefani A. (2023). Band-specific altered cortical connectivity in early Parkinson’s disease and its clinical correlates. Mov. Disord..

[b0085] Conti M., Garasto E., Bovenzi R., Ferrari V., Mercuri N.B., Di Giuliano F., Cerroni R., Pierantozzi M., Schirinzi T., Stefani A., Rocchi C. (2024). Insular and limbic abnormal functional connectivity in early-stage Parkinson’s disease patients with autonomic dysfunction. Cereb. Cortex.

[b0090] Conti M., Bovenzi R., Palmieri M.G., Placidi F., Stefani A., Mercuri N.B., Albanese M. (2024). Early effect of onabotulinumtoxinA on EEG-based functional connectivity in patients with chronic migraine: a pilot study. Headache.

[b0095] De Micco R., Tessitore P.A., Di Nardo F., De Mase A., Giordano A., Caiazzo G., Esposito F., Tedeschi G. (2018). Sex-specific pattern of sensori-motor network connectivity in de novo Parkinson’s disease patients. Eur. J. Neurol..

[b0100] De Micco R., Esposito F., di Nardo F., Caiazzo G., Siciliano M., Russo A., Cirillo M., Tedeschi G., Tessitore A. (2019). Sex-related pattern of intrinsic brain connectivity in drug-naïve Parkinson’s disease patients. Mov. Disord..

[b0105] De Micco R., Agosta F., Basaia S., Siciliano M., Cividini C., Tedeschi G., Filippi M., Tessitore A. (2021). Functional connectomics and disease progression in drug-naïve Parkinson’s disease patients. Mov. Disord..

[b0110] Desikan R.S., Ségonne F., Fischl B., Quinn B.T., Dickerson B.C., Blacker D., Buckner R.L., Dale A.M., Maguire R.P., Hyman B.T., Albert M.S., Killiany R.J. (2006). An automated labeling system for subdividing the human cerebral cortex on MRI scans into gyral based regions of interest. Neuroimage.

[b0115] Fischl B. (2012). FreeSurfer. Neuroimage.

[b0120] Folstein M.F., Folstein S.E., McHugh P.R. (1975). “Mini-mental state”. A practical method for grading the cognitive state of patients for the clinician. J. Psychiatr. Res..

[b0125] Goetz C.G., Poewe W., Rascol O., Sampaio C., Stebbins G.T., Counsell C., Giladi N., Holloway R.G., Moore C.G., Wenning G.K., Yahr M.D., Seidl L. (2004). Movement Disorder Society Task Force report on the Hoehn and Yahr staging scale: Status and recommendations. Mov. Disord..

[b0130] Goetz C.G., Tilley B.C., Shaftman S.R., Stebbins G.T., Fahn S., Martinez-Martin P., Poewe W., Sampaio C., Stern M.B., Dodel R., Dubois B., Holloway R., Jankovic J., Kulisevsky J., Lang A.E., Lees A., Leurgans S., LeWitt P.A., Nyenhuis D., Olanow C.W., Rascol O., Schrag A., Teresi J.A., van Hilten J.J., LaPelle N., Agarwal P., Athar S., Bordelan Y., Bronte-Stewart H.M., Camicioli R., Chou K., Cole W., Dalvi A., Delgado H., Diamond A., Dick J.P., Duda J., Elble R.J., Evans C., Evidente V.G., Fernandez H.H., Fox S., Friedman J.H., Fross R.D., Gallagher D., Goetz C.G., Hall D., Hermanowicz N., Hinson V., Horn S., Hurtig H., Kang U.J., Kleiner-Fisman G., Klepitskaya O., Kompoliti K., Lai E.C., Leehey M.L., Leroi I., Lyons K.E., McClain T., Metzer S.W., Miyasaki J., Morgan J.C., Nance M., Nemeth J., Pahwa R., Parashos S.A., Schneider J.S.J.S., Schrag A., Sethi K., Shulman L.M., Siderowf A., Silverdale M., Simuni T., Stacy M., Stern M.B., Stewart R.M., Sullivan K., Swope D.M., Wadia P.M., Walker R.W., Walker R., Weiner W.J., Wiener J., Wilkinson J., Wojcieszek J.M., Wolfrath S., Wooten F., Wu A., Zesiewicz T.A., Zweig R.M. (2008). Movement Disorder Society-Sponsored Revision of the Unified Parkinson’s Disease Rating Scale (MDS-UPDRS): Scale presentation and clinimetric testing results. Mov. Disord..

[b0135] Grech R., Cassar T., Muscat J., Camilleri K.P., Fabri S.G., Zervakis M., Xanthopoulos P., Sakkalis V., Vanrumste B. (2008). Review on solving the inverse problem in EEG source analysis. J. Neuroeng. Rehabil..

[b0140] Guerra A., Colella D., Giangrosso M., Cannavacciuolo A., Paparella G., Fabbrini G., Suppa A., Berardelli A., Bologna M. (2022). Driving motor cortex oscillations modulates bradykinesia in Parkinson’s disease. Brain.

[b0145] Hall J.E. (2015). Endocrinology of the Menopause. Endocrinol. Metab. Clin. North Am..

[b0150] Hardmeier M., Hatz F., Bousleiman H., Schindler C., Stam C.J., Fuhr P. (2014). Reproducibility of functional connectivity and graph measures based on the phase lag index (PLI) and weighted phase lag index (wPLI) derived from high resolution EEG. PLoS One.

[b0155] Hyvärinen A., Oja E. (2000). Independent component analysis: Algorithms and applications. Neural Netw..

[b0160] Jatoi M.A., Kamel N., Faye I., Malik A.S., Bornot J.M., Begum T. (2016). IEEE 2015 International Conference on Signal and Image Processing Applications.

[b0165] Jwo D.J., Chang W.Y., Wu I.H. (2021). Windowing Techniques, the Welch Method for Improvement of Power Spectrum Estimation. Computers, Materials and Continua.

[b0170] Kompoliti K. (2003). Estrogen and Parkinson’s disease. Front. Biosci..

[b0175] Little S., Brown P. (2014). The functional role of beta oscillations in Parkinson’s disease. Parkinsonism Relat. Disord..

[b0180] Meoni S., Macerollo A., Moro E. (2020). Sex differences in movement disorders. Nat. Rev. Neurol..

[b0185] Moisan F., Kab S., Mohamed F., Canonico M., Le Guern M., Quintin C., Carcaillon L., Nicolau J., Duport N., Singh-Manoux A., Boussac-Zarebska M., Elbaz A. (2016). Parkinson disease male-to-female ratios increase with age: French nationwide study and meta-analysis. J. Neurol. Neurosurg. Psychiatry.

[b0190] Morris R., Martini D.N., Madhyastha T., Kelly V.E., Grabowski T.J., Nutt J., Horak F. (2019). Overview of the cholinergic contribution to gait, balance and falls in Parkinson’s disease. Parkinsonism Relat. Disord..

[b0195] Nasreddine Z.S., Phillips N.A., Bédirian V., Charbonneau S., Whitehead V., Collin I., Cummings J.L., Chertkow H. (2005). The Montreal Cognitive Assessment, MoCA: A brief screening tool for mild cognitive impairment. J. Am. Geriatr. Soc..

[b0200] Nitkowska M., Tomasiuk R., Czyzyk M., Friedman A. (2015). Prolactin and sex hormones levels in males with Parkinson’s disease. Acta Neurol. Scand..

[b0205] Nuwer M.R. (2018). 10-10 electrode system for EEG recording. Clin. Neurophysiol..

[b0210] Okun M.S., Walter B.L., McDonald W.M., Tenover J.L., Green J., Juncos J.L., DeLong M.R. (2002). Beneficial effects of testosterone replacement for the nonmotor symptoms of Parkinson disease. Arch. Neurol..

[b0215] Peter W. (2018).

[b0220] Picillo M., Nicoletti A., Fetoni V., Garavaglia B., Barone P., Pellecchia M.T. (2017). The relevance of gender in Parkinson’s disease: a review. J. Neurol..

[b0225] Picillo M., Lafontant D.E., Bressman S., Caspell-Garcia C., Coffey C., Cho H.R., Burghardt E.L., Dahodwala N., Saunders-Pullman R., Tanner C.M., Amara A.W. (2022). Sex-Related Longitudinal Change of Motor, Non-Motor, and Biological Features in Early Parkinson’s Disease. J. Parkinsons Dis..

[b0230] Postuma R.B., Berg D., Stern M., Poewe W., Olanow C.W., Oertel W., Obeso J., Marek K., Litvan I., Lang A.E., Halliday G., Goetz C.G., Gasser T., Dubois B., Chan P., Bloem B.R., Adler C.H., Deuschl G. (2015). MDS clinical diagnostic criteria for Parkinson’s disease. Mov. Disord..

[b0235] Serin E., Zalesky A., Matory A., Walter H., Kruschwitz J.D. (2021). NBS-Predict: A prediction-based extension of the network-based statistic. Neuroimage.

[b0240] Stefani A., Lozano A.M., Peppe A., Stanzione P., Galati S., Tropepi D., Pierantozzi M., Brusa L., Scarnati E., Mazzone P. (2007). Bilateral deep brain stimulation of the pedunculopontine and subthalamic nuclei in severe Parkinson’s disease. Brain.

[b0245] Tadel F., Baillet S., Mosher J.C., Pantazis D., Leahy R.M. (2011). Brainstorm: A user-friendly application for MEG/EEG analysis. Comput. Intell. Neurosci..

[b0250] van der Zee S., Kanel P., Gerritsen M.J.J., Boertien J.M., Slomp A.C., Müller M.L.T.M., Bohnen N.I., Spikman J.M., van Laar T. (2022). Altered Cholinergic Innervation in De Novo Parkinson’s Disease with and Without Cognitive Impairment. Mov. Disord..

[b0255] Varangis E., Habeck C.G., Razlighi Q.R., Stern Y. (2019). The Effect of Aging on Resting State Connectivity of Predefined Networks in the Brain. Front. Aging Neurosci..

[b0260] Visser M., Marinus J., Stiggelbout A.M., van Hilten J.J. (2004). Assessment of autonomic dysfunction in Parkinson’s disease: The SCOPA-AUT. Mov. Disord..

[b0265] Weis S., Hodgetts S., Hausmann M. (2019). Sex differences and menstrual cycle effects in cognitive and sensory resting state networks. Brain Cogn..

[b0270] Wen X., Wu X., Liu J., Li K., Yao L. (2013). Abnormal Baseline Brain Activity in Non-Depressed Parkinson’s Disease and Depressed Parkinson’s Disease: A Resting-State Functional Magnetic Resonance Imaging Study. PLoS One.

[b0275] Yassine S., Gschwandtner U., Auffret M., Achard S., Verin M., Fuhr P., Hassan M. (2022). Functional Brain Dysconnectivity in Parkinson’s Disease: A 5-Year Longitudinal Study. Mov. Disord..

[b0280] Zalesky A., Fornito A., Bullmore E.T. (2010). Reference Manual for NBS Connectome (v1.2). Neuroimage.

[b0285] Zappia M., Nicoletti A. (2010). The role of the long-duration response to levodopa in Parkinson’s disease, in. J. Neurol..

[b0290] Zhang C., Cahill N.D., Arbabshirani M.R., White T., Baum S.A., Michael A.M. (2016). Sex and Age Effects of Functional Connectivity in Early Adulthood. Brain Connect..

[b0295] Zhang, C., Dougherty, C.C., Baum, S.A., White, T., Michael, A.M., 2018. Functional connectivity predicts gender: Evidence for gender differences in resting brain connectivity. Hum Brain Mapp 39. doi: 10.1002/hbm.23950. 10.1002/hbm.23950 PMC6866578 29322586

